# Supplementary material for: Measuring job satisfaction of midwives: A scoping review
Source: PLoS One. 2022 Oct 13;17(10):e0275327. doi: 10.1371/journal.pone.0275327 (PMC9560034; doi:10.1371/journal.pone.0275327)
Supplement: S1 File — (DOCX) [file pone.0275327.s002.docx]

**S2 File. Exemplary Search String**

Search date: until 20 February 2022

Search String Pubmed

Search (((((((midwifery[MeSH Terms]) OR midwi*[Title/Abstract])) AND (((((hospital) OR obstetric*) OR ward) OR unit) OR department))) AND ((((((((("job satisfaction"[MeSH Terms]) OR "job satisfaction"[Title/Abstract]) OR "job satisfaction") OR "work satisfaction") OR "quality of work life") OR "work experience") OR "employee satisfaction")) AND (((((((((((questionnaire) OR instrument) OR scale) OR "measuring instrument") OR measurement) OR assessment) OR appraisal) OR evaluation) OR survey) OR interview) OR "focus group"))))
