# Supplementary material for: Measuring job satisfaction of midwives: A scoping review
Source: PLoS One. 2022 Oct 13;17(10):e0275327. doi: 10.1371/journal.pone.0275327 (PMC9560034; doi:10.1371/journal.pone.0275327)
Supplement: Attachment — Submitted filename: Response to Reviewers.docx [file pone.0275327.s003.docx]

Sonja Wangler

Tübingerstr. 33

70178 Stuttgart

Germany

Sonja.wangler@dhbw-stuttgart.de

Dear editor, dear peer reviewers,	13.09.2022

we appreciate the opportunity to revise our manuscript. Thank you very much for your peer reviews and appreciative feedback.

Please find our point-by-point response below. We highlighted all changes in the manuscript.

We hope that our manuscript now fulfils the expectations.

| Reviewer 1 | Revision |
| --- | --- |
| Comments to the author: I wondered why the authors did not include the Journal name in Table 2? | Thank you for this recommendation. We now present the journals’ names in Table 2. |
| Reviewer 2 |  |
| Comments to the author: My only suggestion is that more be written on the scoping review method under 'Methods'. | To clarify the method of the scoping review we added “We conducted a scoping review in order to explore the extent of the literature in the field of midwifery job satisfaction and to examine how research in this field is conducted. Scoping reviews aim to identify and map available evidence on an area of research in a transparent way [25-27]. They bring together the evidence from heterogeneous sources and study approaches and can therefore detect research gaps in the existing literature [26, 28].” (see lines 77-81.) |

With kind regards,

Yours sincerely,

Sonja Wangler

on behalf of all co-authors
